# Mangled finger salvage using cross-finger revascularization

**DOI:** 10.1186/s13018-020-01621-w

**Published:** 2020-03-11

**Authors:** Song Chen, Gen Wen, Liang Cheng, Yimin Chai

**Affiliations:** 1 Department of Orthopedics, Shanghai Sixth People’s Hospital East Affiliated to Shanghai University of Medicine & Health Sciences, No. 222 Huanhu Xisan Road, Shanghai, 201306 China; 2 Department of Orthopedics, Shanghai Jiao Tong University Affiliated Sixth People’s Hospital, 600 Yishan Road, Shanghai, 200233 China

**Keywords:** Replantation, Cross-finger revascularization, Microsurgery

## Abstract

**Background:**

Mangled finger with impaired arteria digitalis communis remains to be a challenge for replantation surgery due to the limited amount of tissue to work with.

**Methods:**

Out of 554 hands with total finger amputations treated by replantation of finger/fingers from July 2012 to June 2018, there were 7 cases of damaged arteria digitalis communis, all of which were replanted by anastomosing distal adjacent radial/ulnar digital artery to distal end of ulnar/radial digital artery of amputation finger, and 2 veins were anastomosed for each finger. A skin pedicle was made by suturing both dorsal and palmar skin of adjacent fingers, and detachment was performed 4 weeks postoperatively.

**Results:**

The survival rate was 100%. Mean total active motion was 191.4° (ranging from 170 to 220°). Mean 2-point discrimination was 8 mm static (ranging from 6 to 11 mm), and mean grip strength was 35.3 kg (range, 29 to 40 kg).

**Conclusions:**

Based on our experience, cross-finger revascularization is an effective and safe alternative for mangled finger salvage when arteria digitalis communis is damaged, and good functional prognosis can be expected.

## Background

Komatsu and Tamai reported the first successful finger replantation in 1968 [1]. Over the ensuing four decades, many refinements and advances had been made in microsurgical techniques resulting in a success rate of 92–99% for digital replantation, but replantation surgery is still a challenge because the surgeon needs to perform the operation quickly while potentially be facing limited amount of tissue to work with [2, 3]. Many factors can affect the prognosis, but impaired arteria digitalis communis is a particular problem for finger replantation surgery. Most surgeons treat digital amputation with damaged arteria digitalis communis as a complication of replantation. Both the proximal and distal neurovascular bundles are extensively damaged, making it difficult to estimate the level of tissue injury even with the help of an operating microscope. The aim of this study is to highlight the technical advantage of using cross-finger revascularization in salvage of digits with damaged arteria digitalis communis and the positive functional prognosis this can bring to the table.

## Materials and methods

From July 2012 to June 2018, a retrospective study was conducted on 554 hands with total finger amputations treated by replantation of finger/fingers. Seven of these cases were complicated with damaged arteria digitalis communis and were replanted by anastomosing distal adjacent radial/ulnar digital artery to distal end of ulnar/radial digital artery of amputation finger. Out of the 7 cases, 5 were males and 2 were females, with a mean age of 35.3 years old (ranging from 18 to 55 years old). All of them were work-related injuries. One case was complicated with a type III (according to Urbaniak’s classification [4]) degloving injury in her right hand. All of the 7 cases were followed up for a mean 19.1 months (ranging from 13 to 25 months). Assessments of hand functions were conducted at the end of this study; no other procedures were done before the assessment. One surgeon, who was not involved in the primary treatment of the patients, assessed the patients’ hand functions, including total active motion, grip strength, and 2-point discrimination.

Each patient underwent radiographs of the hand(s), as well as blood routine examination, coagulation function, and electrocardiogram, right after being admitted to emergency room. No systemic diseases that could complicate the healing were reported.

Patients were told to remain in the supine position under brachial plexus anesthesia. The replantation team consists of two groups. One group brings the amputation part to a tagging table in the operation room once the patients arrive in the emergency room. The part is gently debrided with surgical preparation, and the blood vessels and nerves were examined by microsurgical instruments with the aid of operating microscope. Here, the surgeon will determine whether or not the part can be replanted, and whether vein grafting or cross-finger revascularization should be applied. The arteries and venous were tagged with small microvessel sutures. Next, the bone should be trimmed and shortened as needed, and two crossed Kirschner wires were retrograde inserted. Tendons were then traced through the tendon sheaths and tagged with tendon sutures.

**Fig. 1:**
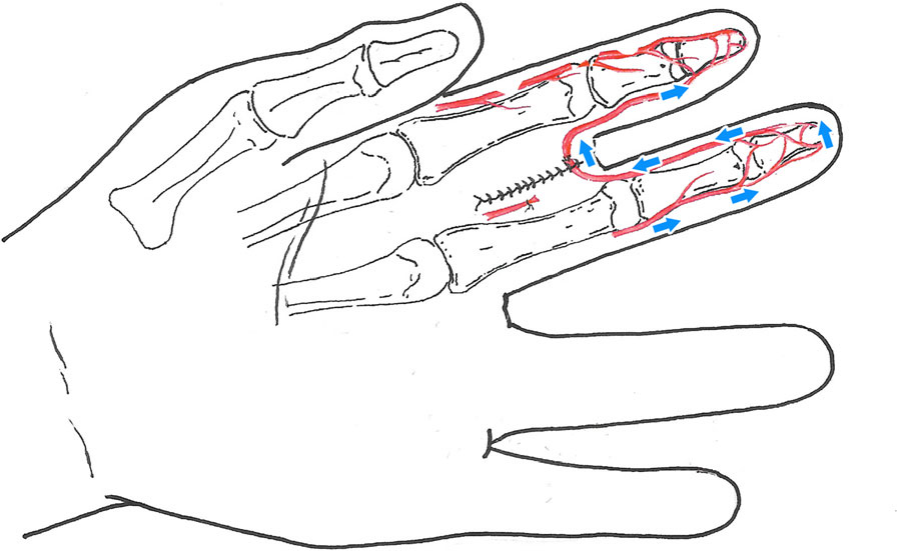
A drawn figure shows the cross-finger revascularization technique and the direction of blood flow

Meanwhile, the proximal part could start being debrided by the other group once the anesthesia was set. The condition of the proximal vascular should be explored from distal to proximal in order to determine the degree of vascular injury, and it is the most important step in the systematic sequence according to individual surgeon’s preference. Therefore, a second longitudinal incision was made at the mid-portion of the middle phalanx to the metacarpophalangeal (MP) joint of the adjacent digit, and the radial/ulnar digit artery was incised at the proximal interphalangeal joint (PIP joint). The arteries were tagged with microvessel clips. Then, the bony fixation and tendon repairs can be performed. The distal radial/ulnar digital artery of the adjacent finger was anastomosed to the distal stump of the ulnar/radial digital artery of the amputated finger. A skin pedicle was made by suturing the dorsal skin of the adjacent finger to the dorsal skin of the amputated finger and the palmar skin of the adjacent finger to the palmar skin of the amputated finger (Fig. 1). Nerve repair was performed if the nerve was not defect. Out of the 7 cases, 2 patients had nerve repaired.

Intraoperative intravenous dextran-40 was injected at the first anastomosis at 25 cc/h. The dextran-40 injection was continued for 5 days postoperatively. The anticoagulation protocols vary, depending on local practices. The hand and waist were elevated on pillows and kept warm. One hundred milligrams of oral acetylsalicylic acid was given for 30 days postoperatively.

All of the 7 cases were followed up for a mean 19.1 months (ranging from 13 to 25 months). Detachments of the amputated finger and adjacent finger were performed after 4 weeks and both fingers showed signs of good blood supply. The K-wires were removed simultaneously during the detachments. Passive functional exercises began right after detachments. Active functional exercise began 2 weeks later postoperatively.

## Results

The mean follow-up period was 19.1 months (ranging from 13 to 25 months). As it is important of the vein treatment in the replantation, 3 to 4 veins were anastomosed in each patient. The total survival rate was 100%. A total of 6 of patients (85.7%) achieved protective sensation of replanted fingers. Mean 2-point discrimination was 8 mm static (ranging from 6 to 11 mm), and mean grip strength was 35.3 kg (ranging from 29 to 40 kg). Mean total active motion was 191.4° (ranging from 170 to 220°). The lengths of the replanted digits compared with contralateral side ranged from 88 to 100% (mean 93.3%). No postoperative vascular complications occurred in our study.

### Case report

**Fig. 2:**
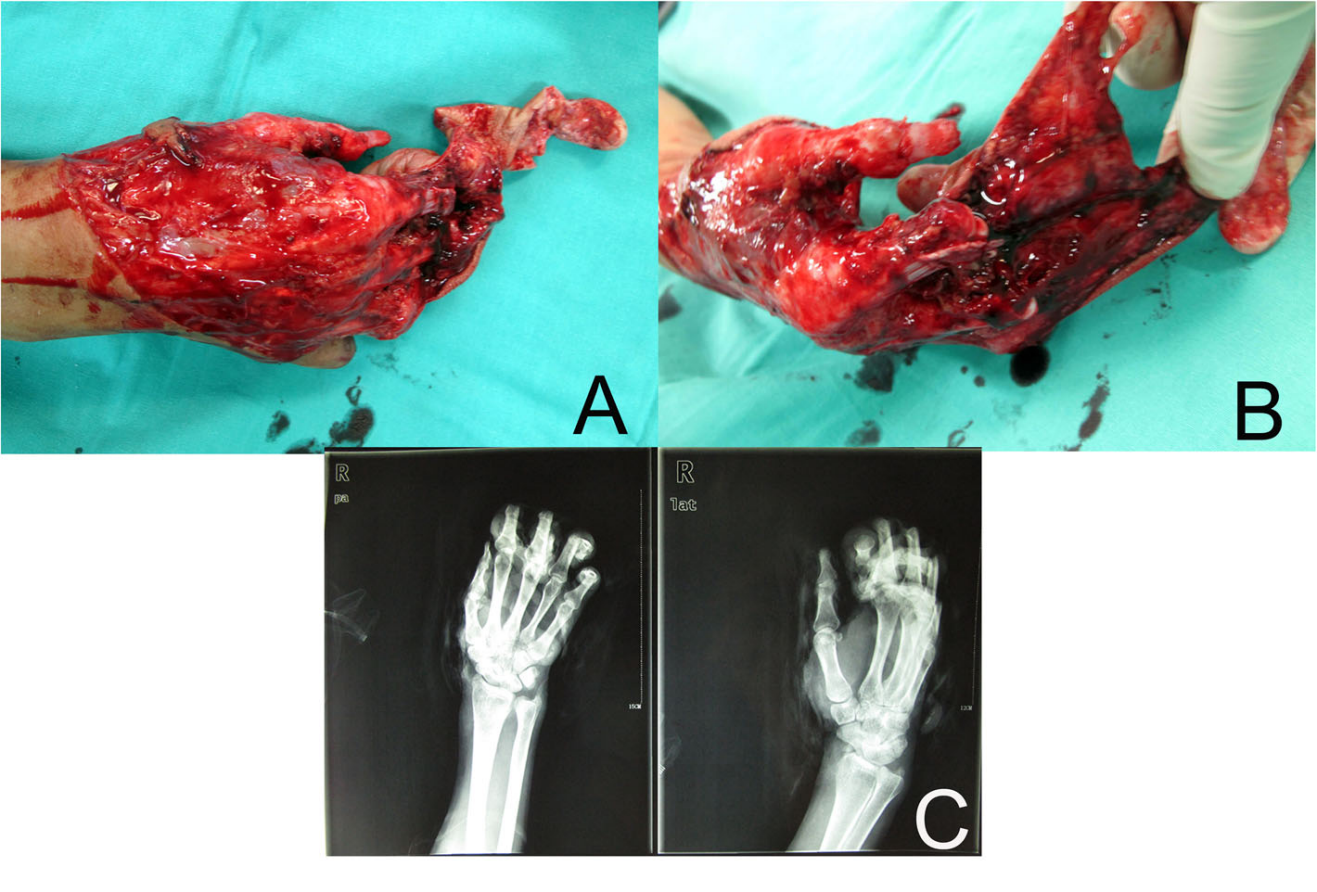
Clinical and radiological examinations of her right hand. **a**, **b** Shows a degloving injury in her right hand with PIP joint dislocation of the patient’s index and middle fingers. **c** Shows the radiological examination of her right hand

A 38-year-old woman presented with a degloving injury in her right hand due to an industrial accident. Clinical and radiological examinations of her right hand showed PIP joint dislocation of the patient’s index and middle fingers. The radial digital artery of her index finger was avulsed from the proximal phalanx, and the distal part of her index finger had no blood flow and was not revascularizable (Fig. 2).

**Fig. 3:**
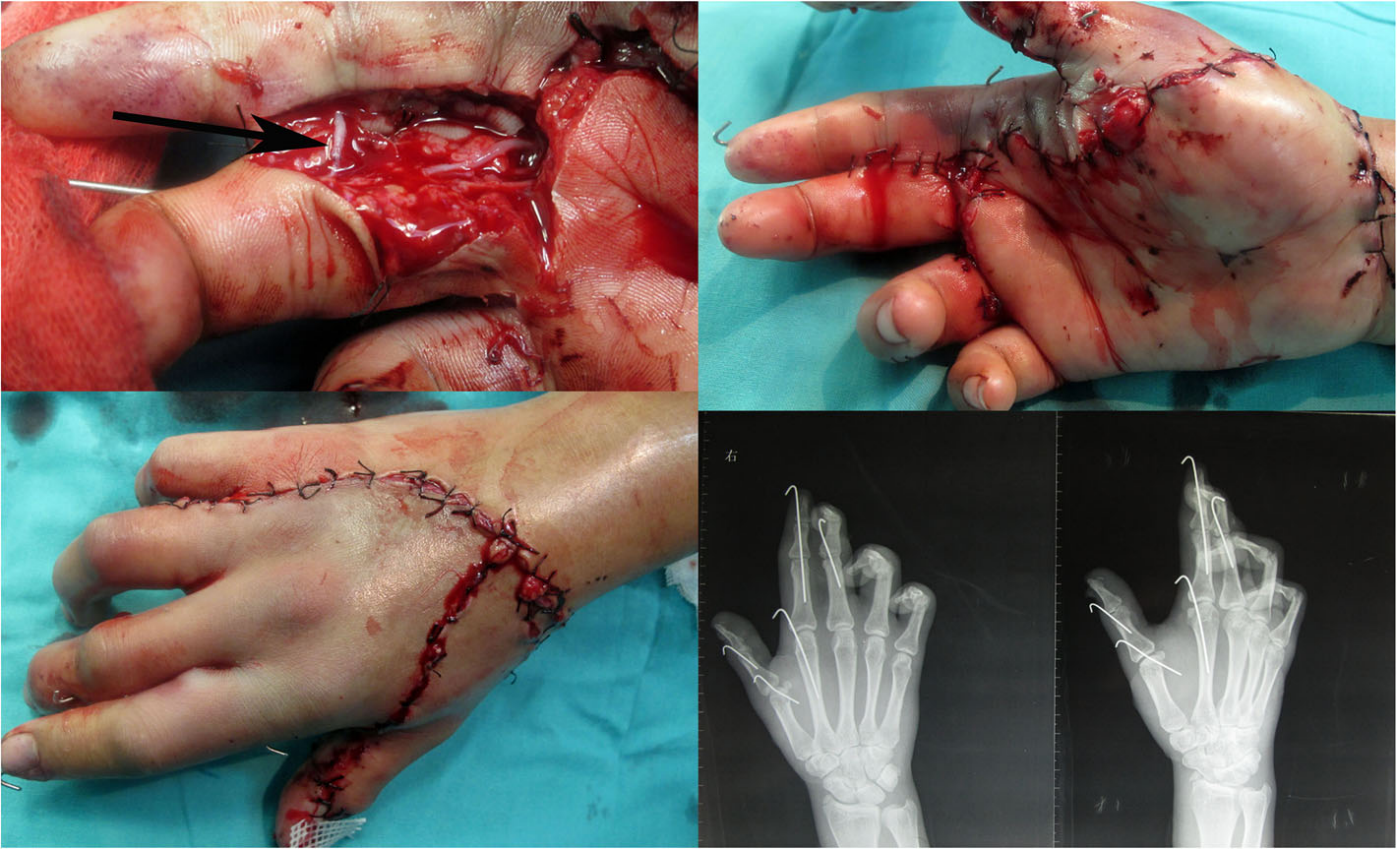
The cross-finger revascularization technique for mangled finger salvage. **a** Shows the degloving skin of her thumb and the dorsal surface of her hand was sutured in situ. **b** Shows the distal radial digital artery of the middle finger was anastomosed to the distal stump of the ulnar digital artery of the index finger. **c** Shows a skin pedicle was made by suturing the dorsal skin of the middle finger to the palmar skin of the index finger

After debridement and reduction of the dislocated PIP and MP joints, a long vein graft was taken from the palmar side of her forearm to reconstruct an outflow course for her thumb and index finger. The degloving skin of her thumb and the dorsal surface of her hand were sutured in situ (Fig. 3a). The ulnar digital artery and nerve of her thumb were repaired to restore blood flow and sensation, respectively.

To restore blood supply to the patient’s index finger, a mid-lateral skin incision was made from the distal interphalangeal joint (DIP joint) to the MP joint. The radial digital artery of the patient’s index finger was severely injured and the proximal part could not be explored. The ulnar digital artery of her index finger was twisted. The incision was extended to the middle of her hand, as the first arteria digitalis communis was also injured. Therefore, a second incision was made at the midportion of the middle phalanx to the MP joint of the middle finger, and the radial digital artery was incised at the PIP joint. The blood flow at the proximal stump of the radial digital artery was not ideal, which confirmed that the first arteria digitalis communis was damaged in the degloving injury. Since the blood flow from the distal radial digital artery of the middle finger was good, the distal radial digital artery of the middle finger was anastomosed to the distal stump of the ulnar digital artery of the index finger. A skin pedicle was made by suturing the dorsal skin of the middle finger to the palmar skin of the index finger. No circulatory problems occurred postoperatively (Fig. 3b, c).

**Fig. 4:**
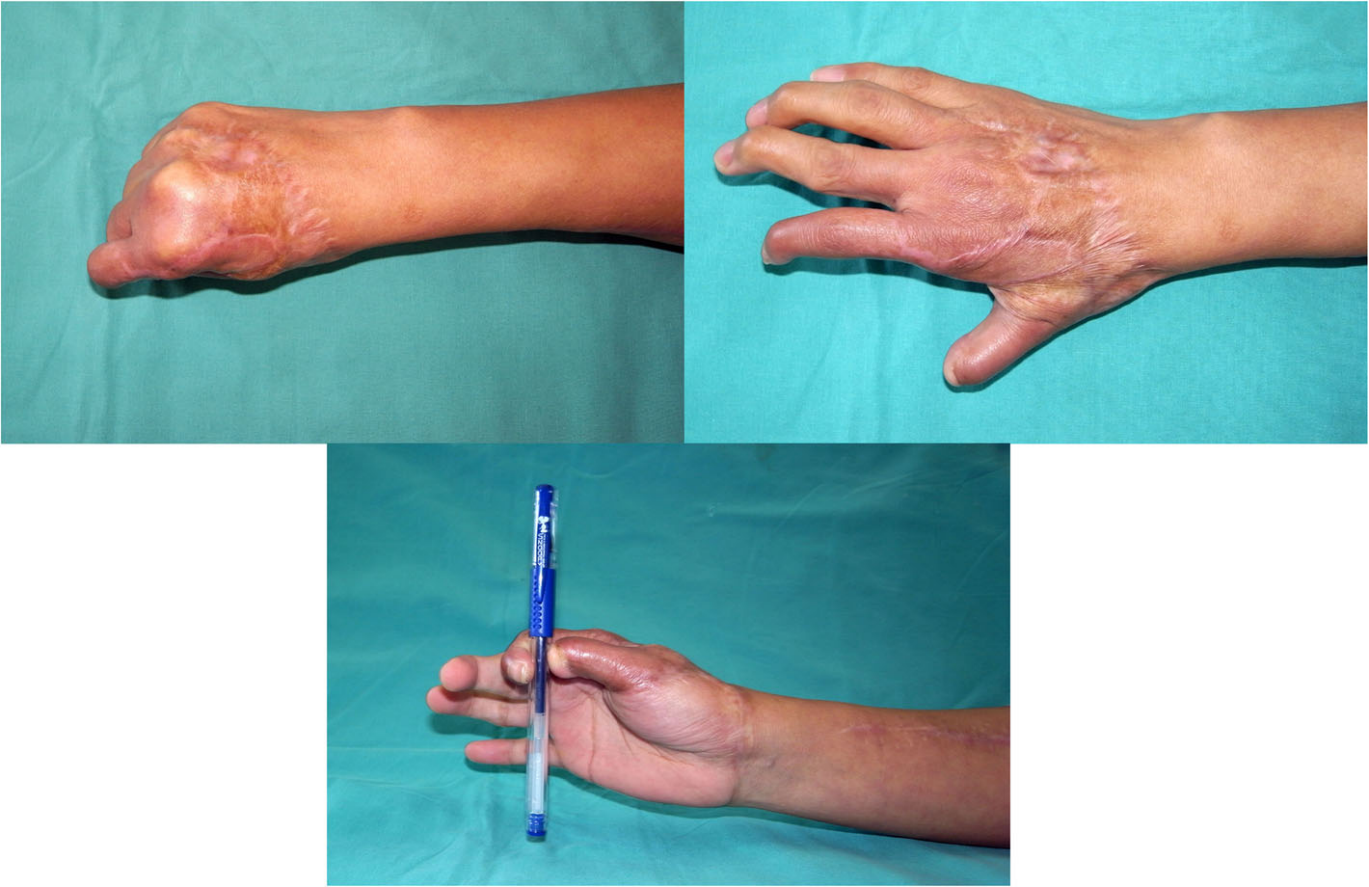
The appearance and function of the index and middle fingers were satisfactory

Detachment of the index and middle fingers was performed 4 weeks after and both fingers showed signs of good blood supplies. The K-wires were also removed during the detachment. The appearance and function of the index and middle fingers were satisfactory 16 months postoperatively (Fig. 4).

## Discussion

With the advent of microsurgery, digital and limb replantation has become a routine technique in most microsurgery centers worldwide. However, the survival rate of replanted digits is 45–65% in crush­avulsion injuries, which is much lower compared to the more common amputations [5]. In avulsion injures, it is often difficult to estimate the degree of tissue injury, even with the help of an operating microscope [5, 6]. Usually, proximal and distal neurovascular bundles are extensively damaged. After thorough debridement, widespread defects of the vascular structures can occur; thus, an end–to-end anastomosis is not suitable.

Many techniques have been applied to correct vascular defects, such as autogenous vein graft transfer and vessel rerouting from neighboring digits. Ozkan et al. used a long vein graft from the volar aspect of distal forearm to bridge the vessel defect in avulsion injuries [7]. The authors advised that careful attention should be paid to preventing any mismatch between the diameter of the vein graft and the vessels [7]. Reducing the length of the amputated proximal stump to compensate for the loss of vasculature is also an effective method. Among the patients in our study, both the radial and ulnar digital arteries of the amputated finger and the arteria digitalis communis were severely injured during the crush injury. However, most of the patients retained uninjured metacarpophalangeal joints or proximal interphalangeal joint, which would achieve good range of motion postoperatively as long as they are salvaged.

In these cases without any suitable vessels, the vein grafting, as backups for replantation attempt, it is difficult to provide sufficient arterial inflow to the traumatized bed [4, 8]. Furthermore, a large discrepancy in vessel size between the vein graft and the distal/proximal end of the artery is a great challenge to the surgeons. So, a syndactylia of the amputated and adjacent fingers was formed by suturing the dorsal skin of the adjacent finger to the dorsal skin of the amputated finger, and the palmar skin of the adjacent finger to the palmar skin of the amputated finger.

The most important functional prognosis is the preservation of finger sensibility. Based on the results of our study, a total of six patients achieved protective sensation, and mean 2-point discrimination was 8 mm static (ranging from 6 to 11 mm). Although Ozcelik et al. observed an average of 7.2 mm static two-point discrimination in 31 replantations with no nerve repair in patients aged 6 to 40 years, and Faivre et al. reported an average of 4.6 mm static two-point discrimination in eight patients younger than 16 years with replantations distal to the distal interphalangeal joint [9, 10]. The results in this study can possibly be explained by the greater degree of spontaneous neurotization in children. Most of the patients in our study are crush injuries, which can cause rupture and contusion of digital nerve. Thus, we performed nerve anastomosis on our patients when necessary.

Preservation of interphalangeal joint range of motion is also an important functional factor of finger replantation, especially since the period of immobilization could result in stiffness of the PIP and MP joints of both replanted and adjacent fingers. Based on our experience, with early range-of-motion exercises after K-wires removal, a good flexion-extension arc over 160°can be expected. Patients in our study have a mean active motion of 191.4° (ranging from 170 to 220°) and mean grip strength of 35.3 kg (ranging from 29 to 40 kg) through active strength exercises.

Another important thing to consider in this study is the patient with degloving injury. Vascular reconstruction of avulsed vessels is not an easy procedure. The survival rate of type III ring avulsion injuries treated by replantation has been reported to range from 73 to 85% [11]. Akuyrek et al. reported a series of 7 patients with type III ring avulsion injuries replanted by radical debridement and vein grafting [12]. As an attempt to increase the survival area of the re-attached skin, four veins on the dorsal and palmar sides were anastomosed respectively. Although the increased number of anastomosed vein might not be a significant indication of survival rate improvement, a large number of anastomosed veins may possibly improve the safety of the procedure if the quality of vessels walls is in question.

## Conclusion

Although there are many other backup methods in the procedure of finger replantation, cross-finger revascularization technique is a good alternative option when both radial and ulnar arteria digitalis communis are badly injured and vein grafting cannot provide sufficient arterial inflow to the replanted finger. However, there are some limitations in this study, the reliability of the cross-finger revascularization technique needs to be further evaluated with a larger number of patients, and the function assessment should be included the adjacent digits in further clinical experiences. Nevertheless, with a comprehensive preoperative planning, this kind of injury has a high success rate, with good functional and aesthetic prognosis. On the other hand, good preoperative communication with the patient and the patients’ family is still necessary.

## Data Availability

The datasets used and/or analyzed during the current study are available from the corresponding author on reasonable request.

## Abbreviations

MR joint: Metacarpophalangeal joint
PIP joint: Proximal interphalangeal joint
DIP joint: Distal interphalangeal joint
